# Human genetics and genomics meetings going virtual: practical lessons learned from two international meetings in early 2020

**DOI:** 10.1186/s40246-020-00275-3

**Published:** 2020-07-08

**Authors:** Alistair R. R. Forrest, Gabriela M. Repetto, Juergen K. V. Reichardt

**Affiliations:** 1 Harry Perkins Institute of Medical Research, QEII Medical Centre and Centre for Medical Research, The University of Western Australia, Nedlands, WA 6009 Australia; 2 Center for Genetics and Genomics, Facultad de Medicina, Clinica Alemana Universidad del Desarrollo, 7710162 Santiago, Chile; 3 Australian Institute of Tropical Health and Medicine, James Cook University, Smithfield, QLD 4878 Australia

**Keywords:** Human genetics, Human genomics, Virtual meeting, Conference

## Abstract

The recent coronavirus disease 2019 (COVID-19) pandemic has caused worldwide disruption which also extends to the arena of scientific meetings around the world. Here, we explore the lessons learned from moving two human genetics and genomics meetings quickly to an online format in early 2020. The tips presented herein may be useful not only for future virtual meetings but may also enrich future physical if not hybrid meetings once they resume.

## Body

Due to the COVID-19 (coronavirus disease 2019) pandemic [https://en.wikipedia.org/wiki/Coronavirus_disease_2019], two recent human genetics and genomics meetings, the Human Genome Meeting (HGM; [http://hugo-hgm2020.org]) and the Global Genomic Medicine Collaborative (G2MC; [https://g2mc.org/genomic-medicine-implementation-in-low-resources-settings-south-america/], https://g2mc.org/events/), pivoted from a traditional physical format to digital delivery. Both meetings are convened annually or biannually under certain circumstances with attendance around 500 and lasting over several days. The 2020 HGM and G2MC were convened virtually in the first few months of 2020 pivoting quickly from the traditional physical setup. The HGM had 505 attendees from 37 countries and all continents. This included 123 registrants that only registered after the announcement that the meeting would go fully virtual. HGM2020 was planned for in its physical version and hence run from Perth, WA, Australia, in early April of 2020. The G2MC was envisaged for Santiago, Chile, in early May 2020 and accordingly run mostly virtually from Santiago as well, with 489 registrants from 50 countries across all continents, more than doubling the original 176 registrants from 20 countries.

Several useful and common insights were garnered from the two meetings which will be important tips for future virtual meetings (Table 1).

**Table 1:** Lessons learned from two genetics and genomics meetings

I. Many of our research community cannot travel for reasons such as family commitments, time commitments, funding, and environmental concerns. For these delegates, there is a strong interest in providing online registration options for those who cannot travel (for many reasons). This is a particularly relevant issue for the community in low- and middle-income countries (LMIC).	
II. Virtual meetings can be more cost-effective.	
III. Live talks are well received but require time zone friendly adjustment of speaking slots or use of prerecords.	
IV. On-demand content is essential for those in different time zones and is valuable for all registrants to catch up on missed talks in parallel sessions and for individuals in LMIC with limitations in high-speed internet access. A period of at least 1 month seems reasonable, and speakers should consent.	
V. Plan for a sufficient number of attendees and use stable platforms which can handle access from all registrants.	
VI. A nimble PCO (professional conference organizer) together with a savvy IT provider who can quickly get on top of any technical issues as they arise are critical.	
VII. When live streaming, live talks, when feasible, are better received than prerecords.	
VIII. Virtual receptions to discuss experiences are a welcome addition for participants to network and discuss their experiences.	
IX. Features that facilitate interactivity (chats, Q&A features, polls) are useful to enhance audience participation and networking. The platforms used allowed for interaction at both the virtual HGM and G2MC.	
X. Create a messaging group for the organizing committee to communicate internally and quickly on issues as they arise to solve them quickly and distribute the workload.	
XI. Sponsors are critical to all meetings and can be offered banners in talks at virtual meetings.	
XII. For early career researchers, online on-demand short video presentations may work well if not better than static PDFs of posters. The online on–demand format frees conference organisers from the time limitations of a traditional meeting.	
XIII. Access to talks on demand is of great interest and a benefit of virtual meetings.	

### The virtual Human Genome Meeting (HGM) in Perth, Australia

The HGM2020 meeting was due to be held physically in Perth, Australia, from April 5–8 2020. In January 2020, there were the first indications that COVID-19 may impact the meeting by preventing delegates from China attending. This then expanded to South Korea, Iran, and Italy and eventually travel to Australia from all countries that was restricted. The decision to go fully online was announced on March 14, 2020, 22 days before the start of the meeting.

The switch to a fully online meeting provided an opportunity to challenge the dogma that scientific meetings must be physical. Organizing committee members arguing the benefits of an online only registration option is often blocked by concerns that online registrations would mean reduced physical registration numbers and that presenters would avoid presenting new unpublished work. The main benefits of an online meeting are that it allow participation on those who cannot travel to a physical meeting (environmental concerns, time commitment, family commitments, funding). These issues disproportionately affect early career researchers, women, and those in poorly funded countries and labs. An additional 123 delegates took advantage of the online only option (63 of these were international registrants). The cost of the virtual 2020 meeting which was planned and originally set up until March 2020 as a traditional physical meeting. Therefore, costs were modestly smaller than the cost of the traditional 2019 meeting. In this context, it is worth remembering that the HGM was planned and organized as a traditional physical meeting until about 1.5 months prior to it.

**Fig. 1:**
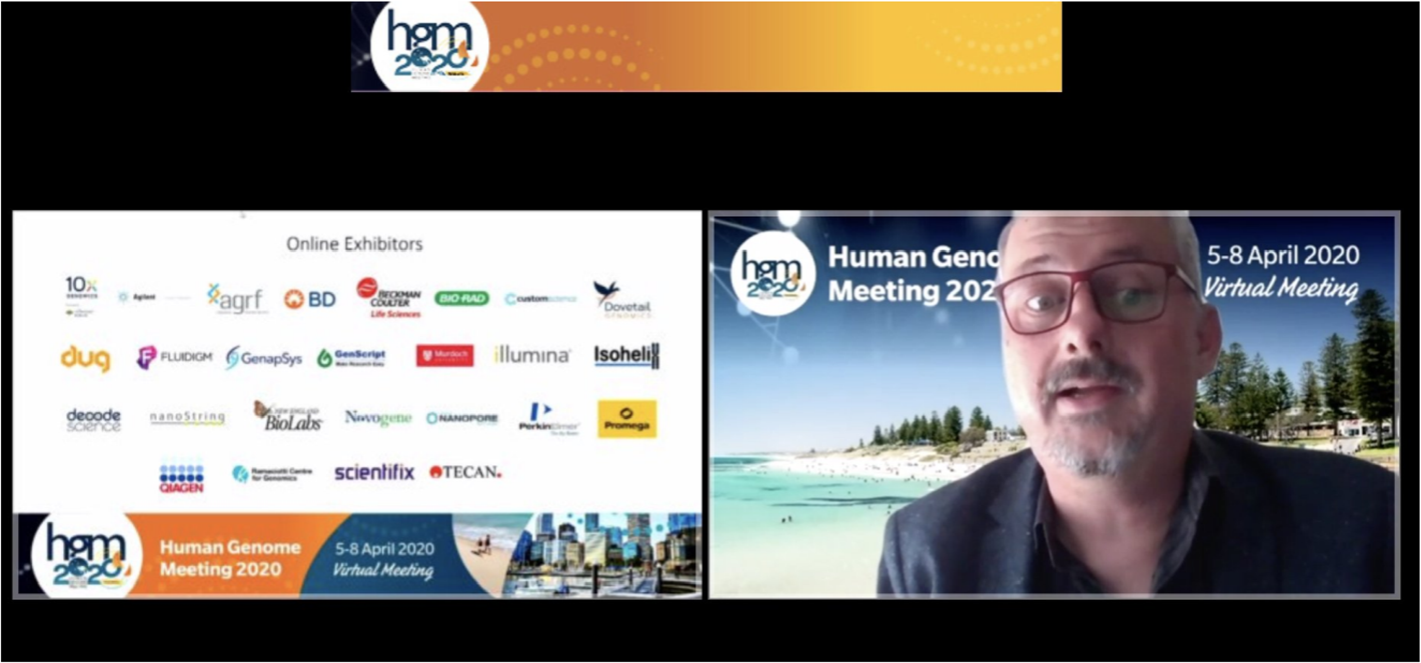
Screenshot of one of the authors (ARRF) presenting at the virtual Human Genome Meeting (HGM) 2020

The virtual HGM included side-by-side slides and speakers presenting which made for an attractive way of viewing talks (Fig. 1).

**Fig. 2:**
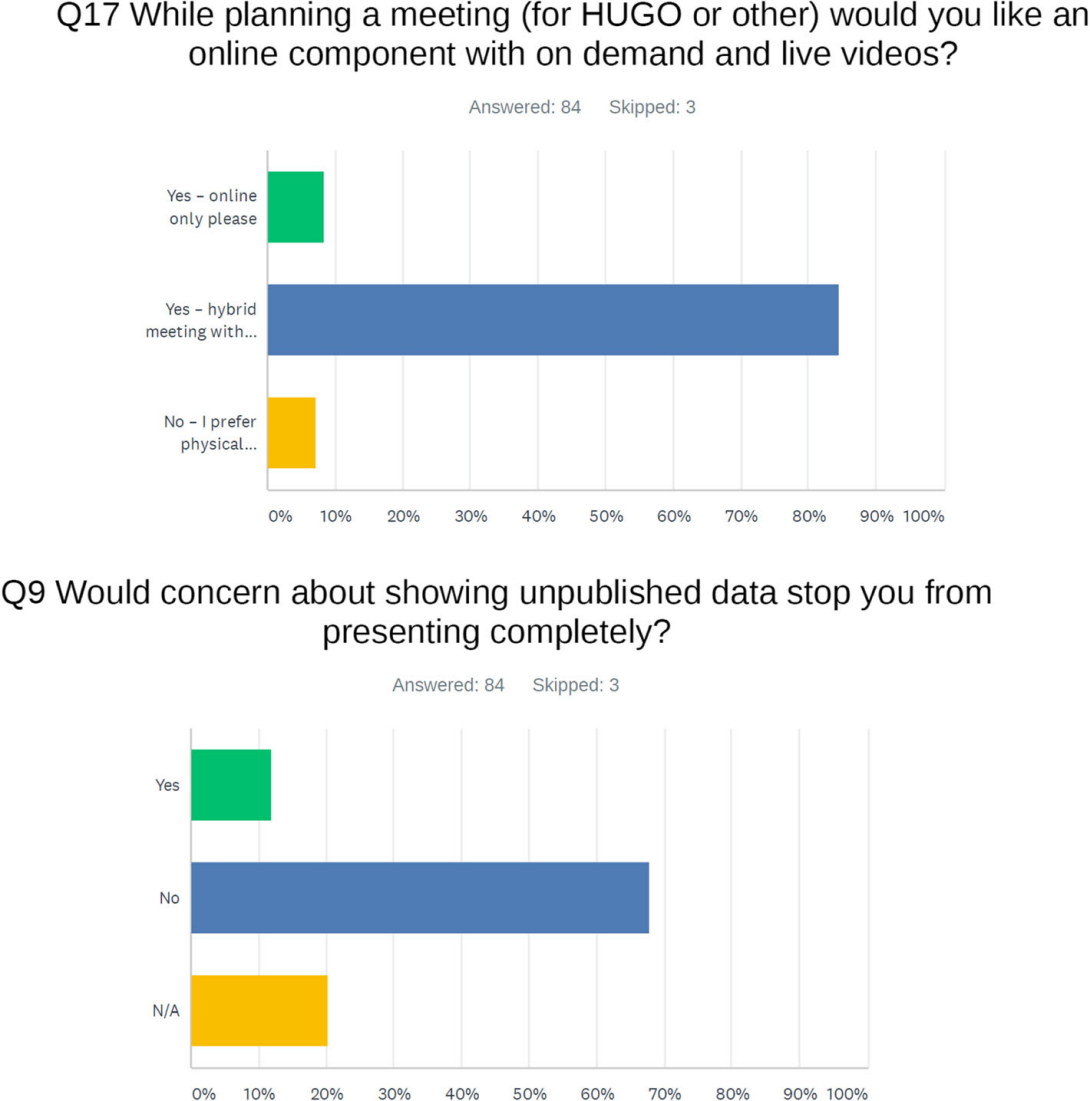
Responses of HGM registrants to the online meeting experience

At the conclusion of the meeting, delegates were asked how they felt the meeting went and whether future meetings should include an online component. Interestingly, when asked whether they would prefer an online only, physical only, or hybrid meeting with both physical and online components, 85% of respondents indicated they would like a hybrid meeting, and only 7% indicated they wanted only physical meetings (Fig. 2). A hybrid meeting format with on-demand video after the meeting is particularly attractive for delegates as it allows them to catch up on content missed due to parallel sessions. For international online only delegates, on-demand video is essential to deal with time problematic time differences.

### The Global Genomic Collaborative (G2MC) meeting

The 5th G2MC meeting was scheduled to occur from May 6–8 2020 in Santiago, Chile [https://g2mc.org/events/]. The theme of the meeting was Genomic Medicine Implementation in Low Resources Settings-Latin America (LATAM), as a follow-up to the previous meeting with a similar focus that took place in Cape Town, South Africa, in December 2018. In early March 2020, given the global epidemiological situation with the first cases of COVID-19 being diagnosed in LATAM, the decision was made to change the meeting to a virtual modality. The program maintained most of its original structure: parallel, highly interactive working-group sessions on specific projects, followed by sequential presentations by invited speakers organized around specific themes. Nevertheless, several changes were introduced: the overall duration of each day´s program was shortened from the original version to accommodate the diverse time zones of the participants, conferences were reduced in duration to focus on highlights of ongoing programs and initiatives, the time allotted to Q&A sessions was increased to promote interactions, and the decision was made to postpone the Young Investigators´ Forum to a future face-to-face meeting to optimize opportunities for networking. The most significant and immediate effect of the virtual format was the increased number and country of origin of registrants, particularly from LMIC, a result that was aligned with the aims and theme of the meeting. Compared to previous G2MC meetings, the increase of registrants from LATAM was noticeable whilst Africa was also again well represented. The G2MC meeting could pivot earlier in the planning to a virtual setting and accordingly realized substantial cost savings. When comparing the costs of past physical and the 2020 virtual meeting, it is important to also bear in mind that both the HGM and the G2MC are held around the world which leads to varying cost structures.

A post meeting survey to participants, 97% declared being “very satisfied or satisfied” with the virtual format. Responders were also satisfied with items related to interaction, such as time allocated for discussion, and opportunities for networking and future collaborations. Over 60% expressed that they would be unable or unsure about participating in a face to face meeting.

## Discussion

Other meetings have also gone virtual to notable success [1] echoing our experiences outlined herein. We assume that upon the end of the COVID-19 pandemic, the structure of scientific meetings will be re-examined. The personal interactions offered by in-person physical meetings may well lead to a resumption of such traditional meetings. However, some form of virtual meetings, e.g. at a reduced rate, may well be worth keeping. Lastly, talks on demand (Table 1; bullet XIII) may also be a popular feature worth carrying forward in all meetings enriching the experience of registrants. Nevertheless, some future human genetics and genomics meetings may also need to prepared to go virtual quickly and our tips may be helpful in that eventuality, too.
